# Leisure-time physical activity and sports in the Brazilian population: A social disparity analysis

**DOI:** 10.1371/journal.pone.0225940

**Published:** 2019-12-03

**Authors:** Margareth Guimarães Lima, Deborah Carvalho Malta, Camila Nascimento Monteiro, Neuciani Ferreira da Silva Sousa, Sheila Rizzato Stopa, Lhaís de Paula Barbosa Medina, Marilisa Berti de Azevedo Barros

**Affiliations:** 1 School of Medical Sciences, State University of Campinas, Campinas, SP, Brazil; 2 School of Nursing, Federal University of Minas Gerais, Belo Horizonte, MG, Brazil; 3 Núcleo de Indicadores e Sistemas de Informação, Hospital Israelita Albert Einstein, São Paulo, SP, Brazil; 4 Department of Preventive Medicine, Medicine School, University of São Paulo, São Paulo, SP, Brazil; 5 Institute of Collective Health, Federal University of Mato Grosso, Cuiabá, MT, Brazil; 6 Department of Health Analysis and Noncommunicable Diseases Surveillance, Health Surveillance Secretariat, Ministry of Health, Brasília, DF, Brazil; Sciensano, BELGIUM

## Abstract

**Objectives:**

To estimate the prevalence of leisure-time physical activity (LTPA) or sports in the Brazilian population according to demographic and income variables.

**Methods:**

Data from 60,202 Brazilian individuals (18 years and over) were analyzed, belonging to the National Health Survey 2013 sample. The prevalence of different modalities of LTPA and sports was estimated according to age, sex, skin color and income. The adjusted prevalence ratios were estimated by Poisson regression.

**Results:**

Of every thousand Brazilians, 695 do not practice LTPA or sports. Walking is the most practiced LTPA (98/1000), followed by soccer (68/1000) and weight training (45/1000). For poor and black men, the most frequent LTPA was soccer, and, for women, gymnastics and walking. The prevalence of weight training and gymnastics was higher for white people compared with black people. All LTPA practices were more prevalent in individuals with higher income, except for soccer. Running on a treadmill and weight training had, respectively, 24.7 and 6.4 times higher prevalence in the richer quartile.

**Conclusions:**

The study allowed identifying the type of LTPA and sport reported as the most frequent by the Brazilian population according to age, sex, skin color, and income, detecting strong social disparities in these practices.

## Introduction

Several studies [1–8] analyzed the levels of leisure-time physical activity (LTPA) based on information about duration and intensity of the practices, and some of them, providing results on the classification of the individuals in active, insufficiently active and inactive. However, fewer studies estimated the prevalence of the type of LTPA practices [9–13].

The guides and recommendations for physical exercise, aimed at promoting health, consider the type of physical activities in addition to levels, duration, frequency and intensity [14,15]. Knowledge on the type of LTPA practiced by the population is important, considering that different dimensions of physical capacity, such as the cardiorespiratory fitness, muscular strength and neuromotor function exert particular influences in the various aspects of health. The best cardiorespiratory fitness may be achieved through aerobic exercises, while increasing muscle strength is possible with weight training exercises and other activities using external weights or the body itself. Exercises as Yoga, Pilates and dance, in their turn, contribute to improve neuromotor functions [14–16].

Each type of LTPA may depend, in a specific way, on some factors for its accomplishment, such as location and income, considering that LTPA can be practiced indoors and paid, or outdoors, in squares, beaches, sidewalks, without necessarily demanding financial resources [17], and some activities require more expensive devices and accessories [18]. Some practices also are dependent on cultural factors [19–21].

Inequities in the health conditions of the population are widely known [22], and studies have shown that the disparities are also present in health behaviors and, particularly, in LTPA [1–5]. Although various studies have been conducted to monitor social disparities in LTPA levels, the analysis regarding type of physical activity are still scarce.

The Brazilian National Human Development Report, published by the United Nations Development Programme (UNDP) [17], showed, based on the National Household Sample Survey (PNAD) data, information on some of the main types of LTPA. Despite having descriptive analyses and not assessing associations regarding race/skin color, it points out to social disparities in the LTPA practice. In addition, the information available in the National Health Survey on the type of the practice of LTPA and sports was not analyzed, and only two population-based investigations assessed the socioeconomic disparities of these practices in the Brazilian population [10,17], but none evaluated them according to race or skin color. Other studies were performed with specific group [23] or in a particular region [24, 25].

The analysis of the disparities regarding type of physical activity may provide knowledge about associations in different directions regarding gender, age, race, income, in the several types of LTPA or sports, expanding and enriching our understanding about more and less widespread practices. These findings will be useful to guide more targeted and specific LTPA strategies.

This study aimed to estimate the prevalence of the type of LTPA or sport reported as the most frequent, and to verify the association with age, sex, skin color and income in the Brazilian population.

## Methods

The study was carried out using data from the PNS, developed by the Brazilian Institute of Geography and Statistics (IBGE) in partnership with the Brazilian Ministry of Health and the Oswaldo Cruz Foundation, in 2013 and 2014. The PNS sampling was stratified and conducted in three stages: census, household, and resident tracts. In the first stage, the primary sampling units (PSU) were composed of one or more census tracts; in the second stage, 10 to 14 households of each PSU were selected randomly; and, in the third and final stage, an adult (18 years or older) in each household was selected with equiprobability among other residents for the individual interview. The number of households covered by the PNS was of 64,348, and 60,202 individuals were interviewed, with 86.1% response rate.

Data were collected using personal digital assistance (PDA), programmed for critic data entry processes at the time of data collection. A detailed description of the development of the PNS was published [26].

Information on LTPA was obtained from 2 questions. The first one was whether the interviewee practiced or not any type of LTPA in the last three months before the survey. If the answer was positive, they were questioned about the LTPA or sport more often practiced in the period and only one of the following was recorded: outdoor walking, treadmill walking, street running, treadmill running, biking (on the street or ergometric), aerobics, swimming, dancing (practicing PA purpose), muscle training, water aerobics, gymnastics/Pilates/stretching/Yoga, soccer, basketball, tennis, martial arts and wrestling, other practices. Each of these activities was analyzed as one variable, categorized in (0) do not practice and 1 (yes).

In order to describe the sample according to the main socio-demographic variables, we also analyzed sex, age in years (18 to 29; 30 to 59; 60 or more), self-reported skin color (black and brown; white), and monthly family income per capita in quartiles: (1) up to R$355.99 (= low income); (2) R$356 to R$677.99; (3) R$678 to 1,199.99; (4) R$1,200.00 or more (= high income).

The prevalence per thousand people of the reported practices was estimated according to age group, sex, skin color and income. Prevalence differences were tested using Pearson’s Chi-square (*Rao-Scott correction*). Multiple Poisson regression models were used to estimate the prevalence ratios (PR) [27] and their respective confidence intervals (CI 95%). We made adjustments for sex and age, considering that these factors can be determinants of LTPA [4, 6, 17, 28]. To verify associations with skin color, we added income to the adjustment in order to know to what extent the relationship between race and type of LTPA can be explained by income, considering the strong income disparity by race [29]. Data analysis was performed using STATA software version 15.0, with *svy* commands, which consider the corrections for non-response and adjustments for the post-stratification [30].

The PNS was approved by the National Research Ethics Committee (CONEP) under process no. 328,159 of June 26, 2013.

## Results

For every 1,000 inhabitants, 695 did not practice LTPA, and, in the elderly group, only 213 for every 1,000 people reported practicing it. The most frequent LTPA was walking (98/1,000), followed by soccer (68/1,000) and weight training (45/1,000). Soccer and muscle training were substantially higher among individuals aged from 18 to 29 years in relation to the elderly. In adults, the activities reported as more frequent were walking, followed by soccer and muscle training. The prevalence of outdoor walking (PR = 2.06) and gymnastics/stretching/Yoga (PR = 1.62) were higher in elderly people, in addition to water aerobics (PR = 26.2), which was rare among younger adults (Table 1).

**Table 1:** Prevalence and prevalence ratios of LTPA and sports reported as the most frequently practiced, according to age group, PNS 2013.

LTPA and sports reported as the most frequently practiced	Prevalence (per thousand inhabitants)					
	Total	Age			Prevalence ratios^a^ adjusted for sex (CI 95%)	
		18 to 29 (1)	30 to 59 (2)	60 or more (3)	2/1	3/1
**No LTPA**	695.2	581.6	718.7	786.8	1.23 (1.20–1.27)	1.34 (1.30–1.38)
**LTPA**						
Outdoor walking	98.1	60.2	106.5	127.0	1.75 (1.54–1.99)	2.06 (1.77–2.39)
Soccer	67.7	149.4	50.0	4.2	0.35 (0.32–0.39)	0.03 (0.02–0.04)
Muscle training	45.1	91.1	35.2	9.2	0.39 (0.34–0.45)	0.10 (0.07–0.14)
Aerobics, spinning, step, jump	17.0	21.6	16.9	10.7	0.76 (0.59–0.99)	0.46 (0.31–0.68)
Street running	14.4	25.7	13.1	1.6	0.52 (0.40–0.68)	0.6 (0.41–0.11)
Gymnastics, stretching, Yoga	14.0	11.6	13.1	20.3	1.09 (0.81–1.48)	1.62 (1.15–2.27)
Biking (street/ergometric)	9.7	11.3	10.5	5.2	0.97 (0.70–1.35)	0.49 (0.32–0.76)
Treadmill walking	7.4	5.6	8.7	6.2	1.55 (1.03–2.32)	1.08 (0.61–1.94)
Martial arts and wrestling	4.5	9.2	3.3	1.3	0.37 (0.23–0.59)	0.15 (0.1–0.45)
Water aerobics	4.3	0.04	3.8	11.4	9.26 (3.05–28.0)	26.2 (8.57–80.2)
Swimming	3.2	4.3	3.0	2.1	0.72 (0.44–1.15)	0.51 (0.26–1.01)
Basketball, volleyball	2.9	6.7	1.9	0.8	0.28 (0.17–0.47)	0.12 (0.03–0.44)
Dancing (practicing PA purpose)	2.7	3.8	2.4	1.9	0.62 (0.37–1.06)	0.48 (0.24–0.98)
Treadmill running	2.6	2.3	3.2	1.9	1.26 (0.73–2.16)	0.79 (0.30–2.14)
Tennis	1.8	1.7	2.2	0.7	1.36 (0.39–4.72)	0.44 (0.1–2.1)
Others	9.0	13.3	7.2	8.4	0.55 (0.37–0.80)	0.64 (0.41–1.01)

^a^ Reference category: 18 to 29 years old

The main LTPA practiced by men were soccer, walking, muscle training, street running and biking. Among women, the most frequent were walking, water aerobics, muscle training, gymnastics and treadmill walking. Regarding sex, the greater differences were in the following practices: soccer (PR = 0.03), martial arts (PR = 0.25) running on the street (PR = 0.31), biking (PR = 0.41), swimming (PR = 0.45) and muscle training (PR = 0.71), with lower frequencies of practice for women. On the other hand, practices markedly more frequent among the female population were water aerobics (PR = 6.23), aerobics (PR = 5.69) and other gymnastics (PR = 4.41), and dancing, which was almost three times higher in this population (Table 2).

**Table 2:** Prevalence (per 1,000) and prevalence ratios of LTPA and sports reported as the most frequently practiced, according to sex, PNS 2013.

LTPA and sports reported as the most frequently practiced	Sex		p	Prevalence ratios adjusted for age^a^ (CI 95%)
	Male	Female		
**No LTPA**	644.9	740.0	<0.0001	1.14 (1.12–1.16)
**LTPA**				
Outdoor walking	76.6	117.2	<0.0001	1.50 (1,36–1.66)
Soccer	138.7	4.5	<0.0001	0.03 (0.02–0.04)
Aerobics, spinning, step, jump	5.0	27.8	<0.0001	5.69 (4.29–7.56)
Street running	22.8	6.9	<0.0001	0.31 (0.23–0.42)
Muscle training	54.4	36.8	<0.0001	0.71 (0.62–0.81)
Gymnastics, stretching, Yoga	5.0	22.1	<0.0001	4.41 (3.30–5.89)
Biking (street/ergometric)	14.2	5.8	<0.0001	0.41 (0.31–0.55)
Treadmill walking	4.7	10.0	0.0001	2.14 (1.47–3.11)
Martial arts and wrestling	7.5	1.8	<0.0001	0.25 (0.15–0.42)
Water aerobics	10.8	71.8	<0.0001	6.23 (3.22–12.04)
Swimming	4.5	2.0	0.0001	0.45 (0.29–0.69)
Basketball, volleyball	2.7	3.2	0.5285	1.24 (0.76–2.02)
Dancing (practicing PA purpose)	1.4	3.9	0.0001	2.77 (1.66–4.63)
Treadmill running	2.1	3.1	0.1196	1.45 (0.91–2.41)
Tennis	3.3	0.4	<0.0001	0.12 (0.04–0.32)
Others	10.9	7.4	0.0229	0.69 (0.49–0.97)

^**a**^ Reference category: Male

All LTPA and sports were more prevalent among individuals who self-reported being white, except for dancing, tennis, martial arts and wrestling, for which no statistically significant difference was observed. Soccer was the only one whose prevalence was higher among black and brown people. After adjustment according to monthly income per capita, associations remained significant only for muscle training and gymnastics (exercises, stretching and Yoga), with a prevalence that was 17% and 39% higher in white people in relation to black, respectively. Soccer remained associated with higher prevalence in black people after adjustment (Table 3).

**Table 3:** Prevalence (per 1,000) and prevalence ratios of LTPA and sports reported as the most frequently practiced, according to skin color, PNS 2013.

LTPA and sports reported as the most frequently practiced	p	PR^a^ (CI 95%)	PR^b^ (CI 95%)^c^
				Black and brown	White
**No LTPA**	718.0	672.8	<0.0001	0.92 (0.91–0.94)	1.00 (0.98–1.02)
**LTPA**					
Outdoor walking	89.9	106.0	0.0005	1.13 (1.03–1.24)	0.99 (0.90–1.09)
Soccer	80.0	54.6	<0.0001	0.79 (0.71–0.88)	0.94 (0.75–0.94)
Muscle training	36.8	53.3	<0.0001	1.62 (1.41–1.86)	1.17 (1.02–1.34)
Street running	12.4	16.5	0.0168	1.48 (1.17–1.88)	1.05 (0.81–1.37)
Aerobics, spinning, step, jump	14.3	20.0	0.0013	1.44 (1.17–1.76)	1.05 (0.86–1.28)
Gymnastics, stretching, Yoga	9.6	18.8	<0.0001	1.89 (1.46–2.44)	1.39 (1.07–1.82)
Biking (street/ergometric)	8.6	10.9	0.0890	1.33 (1.00–1.77)	1.16 (0.86–1.56)
Treadmill walking	4.9	10.1	<0.0001	2.02 (1.43–2.83)	1.29 (0.90–1.87)
Martial arts and wrestling	4.8	3.9	0.4211	0.96 (0.61–1.51)	0.75 (0.47–1.20)
Water aerobics	30.7	57.3	0.0010	1.58 (1.09–2.30)	1.06 (0.72–1.57)
Swimming	2.3	4.2	0.0050	1.94 (1.25–3.01)	1.26 (0.84–1.89)
Basketball, volleyball	2.4	3.6	0.1247	1.67 (1.01–2.77)	1.39 (0.84–2.30)
Dancing (practicing PA purpose)	2.5	2.8	0.6595	1.15 (0.71–1.87)	0.87 (0.53–1.44)
Treadmill running	20.1	34.5	0.0329	1.75 (1.05–2.91)	1.12 (0.67–1.84)
Tennis	1.1	2.5	0.1692	2.30 (0.77–6.86)	1.27 (0.36–4.49)
Others	7.2	10.7	0.0216	1.56 (1.12–2.18)	1.18 (0.83–1.68)

^a^ Prevalence ratio with adjustments by sex and age

^b^ Prevalence ratio with adjustments by sex, age and income

^c^ Reference category: black and brown.

The prevalence of all LTPA practices, reported as the most frequent, was higher in individuals with higher income, comparing the first and last income quartiles, except for soccer. The greatest disparities were observed in treadmill running (PR = 24.7), treadmill walking (PR = 7.6), and muscle training (PR = 6.4). Gymnastics showed a growing gradient as income increased. Soccer was the only practice with higher prevalence in the poorest quartile of the population (Table 4).

**Table 4:** Prevalence (per 1,000) and prevalence ratios of LTPA and sports reported as the most frequently practiced, according to monthly family income per capita, PNS 2013.

LTPA and sports, reported as the most frequently practiced	Income in deciles of minimum wage				PR adjusted by sex and age (CI 95%)^a^		
	1^st^ quartile ^(low income)^ (1)	2^nd^ quartile ^(middle-low income)^ (2)	3^rd^ quartile ^(middle-high income)^ (3)	4^th^ quartile ^(high income)^ (4)			
	Prevalence per 1,000 inhabitants				2/1	3/1	4/1
**No LTPA**	790.6	746.2	709.1	555.8	0.93 (0.91–0.95)	0.86 (0.84–0.88)	0.68 (0.66–0.70)
**LTPA**							
Outdoor walking	67.9	86.5	96.9	135.7	1.25 (1.07–1.46)	1.33 (1.15–1.52)	1.87 (1.63–2.13)
Soccer	85.8	71.8	65.4	51.3	0.92 (0.80–1.06)	0.97 (0.83–1.12)	0.81 (0.69–0.95)
Muscle training	17.0	31.7	45.2	80.6	2.05 (1.52–2.77)	3.34 (2.48–4.50)	6.35 (4.84–8.32)
Street running	6.5	9.8	9.8	29.7	1.64 (0.93–2.88)	1.85 (1.08–3.16)	5.88 (3.54–9.78)
Aerobics, spinning, step, jump	7.6	12.2	17.0	29.3	1.68 (1.13–2.52)	2.50 (1.78–3.50)	4.35 (3.13–6.06)
Gymnastics, stretching, Yoga	5.9	10.0	11.0	27.4	1.68 (1.03–2.73)	1.78 (1.14–2.80)	4.44 (2.94–6.71)
Biking (street/ergometric)	5.8	9.1	1.0	1.3	1.7 (1.07–2.48)	1.93 (1.23–3.02)	2.49 (1.61–3.83)
Treadmill walking	2.3	2.4	7.0	1.7	1.07 (0.53–2.17)	3.13 (1.59–6.16)	7.58 (3.97–14.48)
Martial arts and wrestling	1.7	5.2	3.5	7.0	3.32 (1.62–6.80)	2.60 (1.36–4.96)	5.56 (3.16–9.80)
Water aerobics	1.2	1.6	3.8	9.9	1.12 (0.46–2.79)	2.08 (0.91–4.76)	5.79 (2.84–11.81)
Swimming	0.5	1.4	2.9	7.3	2.62 (0.89–7.70)	5.59 (1.96–15.95)	14.4 (5.40–38.6)
Basketball, volleyball	2.1	3.2	1.9	4.5	1.70 (0.84–3.43)	1.18 (0.63–2.20)	2.95 (1.69–5.14)
Dancing (practicing PA purpose)	1.2	1.5	3.8	4.0	1.29 (0.50–3.30)	3.49 (1.47–8.32)	3.76 (1.69–8.37)
Treadmill running	0.3	1.7	0.2	5.9	6.80 (2.54–18.19)	9.78 (3.81–25.07)	24.70 (11.04–55.27)
Tennis	0.6	0.03	0.5	5.7	0.55 (0.04–0.75)	0.81 (0.08–8.84)	10.19 (1.22–84.95)
Others	3.9	5.4	9.7	15.9	1.45 (0.88–2.39)	2.83 (1.71–4.69)	4.69 (3.02–7.27)

^a^ Reference category: 1st income quartile.

## Discussion

The prevalence of LTPA in this study was low, considering that only 305 out of 1,000 individuals reported practicing LTPA and sports and that this observation is even more alarming among the elderly. Women are 14% less active than men and studies in Brazil [6, 17] and in other countries [4,28] also pointed out to a higher prevalence of LTPA among men and younger individuals. In the Brazilian population, this evidence has been attributed to cultural aspects, considering that boys used to be more encouraged to participate in outdoor games. In the Brazilian culture, girls are most encouraged to engage in children games such as playing house and cooking, generally with dolls [31], stimulating the permanence of women at home in a sexist way.

Walking was the main practice reported, which was also verified by other authors [7, 17, 32], however, some groups of the population, such as men and younger individuals, did not report this activity as the most frequent. The prevalence of walking grew as age increased, similar to what was observed in a study using the PNAD data in 2015 [17]. Although it is a very common practice, national and abroad, the incentives to walking for leisure purposes should be valued and maintained since this exercise can be practiced by different population groups, with low cardiorespiratory and neuromotor demand and minimum cost because it does not require special equipment or accessories, and in democratic places [33]. In addition, special walking programs, such as trekking would be encouraged, which can also attract younger people and men. Brazil has a great potential to the development of these activities. Based on *Camino de Santiago de Compostela*, in Spain, there are three important routes in Brazil, in the states of Minas Gerais and São Paulo: *O Caminho do Sol*, *O Caminho da Fé*, *O Caminho da Luz* [33].

Soccer is the second most frequently practice sport nationwide, but the first among men (139 among 1,000). In Brazil, soccer has always been the favorite sport among men, with great cultural appeal and traditionally practiced in open and public spaces, in the cities, outskirts and neighborhoods, country towns and rural areas [10]. However, VIGITEL data showed a fall in the soccer practice after 2012 in Brazilian capitals and, according to Rodrigues (2008) [34], the decrease of the soccer game in major cities must be due to real estate speculation and the replacement of these spaces with buildings and roads.

Brazil has stood out in women’s soccer, and a female player from the Brazilian team is the athlete who scored the most goals in the history of both women's and men's soccer cups. Nevertheless, the visibility of women in soccer in Brazil is still low, which is mainly due to the prejudice against women participating in this sport, considered for men [19,35]. Female soccer could be a great opportunity in LTPA practices and a good strategy to raise the women’s level of physical activity if it was more encouraged among the female population.

Muscle training is the third LTPA reported and is more prevalent in young adults and men, noting that only 37 women and 9 elder people out of 1,000 individuals reported this practice as the most frequent. One emphasizes that muscle strength and resistance exercises are very important, especially for these population groups. Evidence shows that these activities can act positively on bone and muscular mass, being constituted of considerable exercises to prevent and control musculoskeletal diseases, such as osteoporosis [36, 37]. Muscle strength and resistance exercises, along with balance exercises, also assist in the prevention of falls and disabilities [15,38].

Water aerobics is a very common practice for the elderly in Brazil [21]. This phenomenon can lead to some generation conflict in LTPA, as young and adult people tend to avoid this practice because of prejudice and the elderly, on the other hand, lose the company of younger people.

In this study, the prevalence of the practice of almost all types of LTPA was greater, except for soccer, in individuals with white skin. However, after income adjustments, associations lost significance in most activities. In Brazil, the strong economic inequality in relation to race/skin color is known, disfavoring black people [29], and worse economic conditions tend to hinder the practice of some types of LTPA due to the need for expenses to practice them. In addition, people in worse economic condition usually also live in areas with less facilities and spaces for recreational physical activity. However, the associations between race/skin color and muscle training and gymnastics (localized exercises, stretching and Yoga) remain with higher prevalence among white people, even after adjustments by income, showing that, not just income, but other aspects related to segregation can contribute to the racial disparities in LTPA. Soccer also remains associated with black people and is more prevalent among them. As far as is known, no studies evaluated the association of race/skin color with the practice of several types of LTPA. Although scarce, investigations have only examined walking. Bates et al. (2005) [11], and Kruger et al. (2008) [33] verified a higher prevalence of walking in non-Hispanic white people when compared with black people.

The results of the analyses by income are coherent with the results of the PNAD in 2015, published in a UNDP report [17]. In Brazil, De Sá et al. (2014) [10] observed that the prevalence of muscle training and running are around 3 times higher for people with the better educational level, than for the less educated. It is noteworthy that this study detected a prevalence of treadmill running 24 times higher compared with the extreme income quartiles; treadmill walking was 7.6 times higher, and muscle training was 6.4 times higher in the segment of the highest income. Muscle training, as well as gymnastics, demand equipment and special accessories, which might hinder and decrease its practice among the poorest. Some municipalities have implemented outdoor academies, which include the fixing of exercise equipment for strength training and stretching in open and public places. This could contribute to decrease the disparities, however, the results of these strategies are still little studied [39].

In 2011, the Brazilian Ministry of Health created the Health Academy Program (PAS), which consist of activities integrated to the primary health care of the Brazilian Unified Health System (SUS), in order to promote physical activities, healthy eating and educational programs by territories [40]. Maintaining and analyzing this government strategy is important, since social disparities are still strong in several LTPA. The appropriation of the city, as well as of the public spaces, connects to aspects related to the promotion of health habits and healthy lifestyles and the regular practice of physical activities. Historically, cities have been built as spaces for production of disparities, since they have not been able to meet socially determined specificities [41].

Soccer is a strong social marker, being the only sport more present in the poor and black population and strategies to maintain and not to hinder this practice among this population is important to guarantee some activity for more socioeconomic vulnerable groups. According to the results of the 2015 PNAD, people with lower income reported practicing LTPA especially because they like to compete and to have fun [17], which is contemplated by a soccer match. On the other hand, individuals in the higher-income stratum mostly reported practicing LTPA or sports to improve their quality of life and well-being [17].

The results of this study bring important elements to understand existent disparities in prevalence of the type of LTPA or sport and to provide knowledge about more elite and more democratic practices, pointing out to the need for specific interventions to rise and maintain physical activity levels in the Brazilian population.

However, the study has some limitations. The question about physical activity was self-referred and tend to be overestimated [42, 43], the validity and reliability of the outcome questions are not known, however other studies assessed the LTPA using similar information [9,10,14]. In addition, self-reported data about the most frequent physical LTPA and sports practiced are self-referred and, possibly, the study could be influenced by some information bias. Also, only the most frequently practiced LTPA was analyzed instead of all the activities practiced by the individuals. Nonetheless, describing population subgroups that refer certain practice as the main one was possible. On the other hand, the study was elaborated with data from the PNS, a survey developed with representative sample of the Brazilian population. Information was discussed on the type of LTPA, which have been little analyzed in the national scope, especially focusing on social disparities, which are crucial when considering the population’s health.

## Conclusions

This study allowed identifying the types of LTPA and sports reported as the most frequent by the Brazilian population according to age, sex, race/skin color and income, detecting strong social and demographic disparities in these practices. These results reinforce the importance of encouraging the practice of LTPA, disseminating knowledge on the benefits of the different practices and facilitating these activities through appropriate spaces and means to perform them. These tasks should be maintained, assessed and regularly rethought considering especially the health of the less favored subgroups. In addition, thinking about strategies to try to avoid age and gender prejudice in the types of LTPA is important.
