# Correction: Leisure-time physical activity and sports in the Brazilian population: A social disparity analysis

**DOI:** 10.1371/journal.pone.0228095

**Published:** 2020-01-15

**Authors:** Margareth Guimarães Lima, Deborah Carvalho Malta, Camila Nascimento Monteiro, Neuciani Ferreira da Silva Sousa, Sheila Rizzato Stopa, Lhaís de Paula Barbosa Medina, Marilisa Berti de Azevedo Barros

The first author’s initials are indexed incorrectly in the article XML. The correct initials are Lima MG. The correct citation is: Lima MG, Malta DC, Monteiro CN, da Silva Sousa NF, Stopa SR, Medina LdPB, et al. (2019) Leisure-time physical activity and sports in the Brazilian population: A social disparity analysis. PLoS ONE 14(12): e0225940. https://doi.org/10.1371/journal.pone.0225940
